# Promoting autophagy to mitigate coronavirus disease pathology in the elderly

**DOI:** 10.1002/ctd2.68

**Published:** 2022-05-23

**Authors:** Abby Lund Da Costa, Het Mehta, Ramkumar Mathur

**Affiliations:** ^1^ Department of Geriatrics, School of Medicine and Health Science University of North Dakota Grand Forks USA

## Abstract

In this commentary, we highlight autophagy's important function, while identifying potential therapeutic targets for severe acute respiratory syndrome coronavirus 2 (SARS‐CoV‐2) in the elderly. Autophagy's decline in the elderly causes increased cell senescence and a dysregulated immune system. As this demographic often faces decreased vaccine‐provided immunity, coronavirus disease 2019 treatments must be developed. We discuss a recent study by Acharya et al. (2022) that found that SF2523 induced autophagy, reducing SARS‐CoV‐2 replication. Furthermore, across varying dosages, SF2523 was shown to have a synergistic effect with remdesivir or MU‐UNMC. Consequently, we believe that SF2523, alone or with other anti‐virals, is a promising potential therapeutic for preventing SARS‐CoV‐2‐related mortalities.

Coronavirus disease 2019 (COVID‐19)’s appearance has sparked a global pandemic. When severe acute respiratory syndrome coronavirus 2 (SARS‐CoV‐2) emerged and began to spread worldwide, knowledge of the new virus was limited. Now, roughly two years after the world shut down, leading researchers are still studying the virus and working to develop a vaccine capable of combating the growing number of variants, including Delta, Omicron, BA.2, and Deltacron. One thing has remained clear throughout the emergence of the different variants of COVID‐19: the elderly demographic appears to be among the most affected by COVID‐19 infection. This potentially links back to autophagy, a critical cellular process, which occurs in response to nutrient deprivation and is important to the degradation and removal of harmful cargo, including damaged cellular materials and viral particles, protecting individuals from disease.^1^ The autophagy process declines in the elderly, resulting in increased cell senescence and a deregulated immune system.^2^, ^3^ Additionally, this population is susceptible to decreased vaccination responses.^4^ While the development of a vaccine normally takes 12–18 months, repurposing or identifying currently existing drugs may assist in hastened identification of innovative COVID‐19 therapeutics. To better serve the elderly, promising treatments against COVID‐19 must be developed in the case of decreased vaccine‐provided immunity.

In the early phases of SARS‐CoV‐2 infection, senescent cells can contribute to an uncontrolled cytokine storm and inflammatory response, which can be fatal. Accordingly, COVID‐19 patients with severe cellular senescence face lung failure as well as multi‐tissue dysfunction.^5^ Cellular senescence promotes a hyperimmune inflammatory response and high mortality in the elderly. In aged patients, a large surge of interleukin (IL)6, IL1b, IFN, C‐reactive protein, and tumour necrosis factor ‐ senescence leads to less airway and lung damage and a greater risk of COVID‐19.^6^ Given the proclivity of senescent cells for higher protein synthesis, increased senescence‐associated secretory phenotype inflammatory mediators would make senescent cells an attractive host target for increased viral replication. Interestingly, the loss in autophagy has been described in ageing and ageing‐related disorders, raising concerns about whether the decline in autophagy is involved in COVID‐19 infection. Nonetheless, the role of autophagy in viral infection surveillance has been researched extensively. The interaction between the virus and the host cell has an impact on viral responses mediated by autophagy. Therefore, activating or targeting the PI3Kinase/Akt pathway, as well as stabilizing Beclin, may be effective in inhibiting SARS‐CoV‐2 replication. Reduction of SARS‐CoV‐2 replication and its developing variants of concern (VOCs), such as Delta and Omicron, has been established in a recent study using SF2523, a dual small molecule inhibitor of the PI3K‐/mTOR/BRD4 pathways.^7^ The inhibition of catabolic processes, such as autophagy, entails activating the rapamycin mechanistic target (mTOR). It has been discovered that SARS‐CoV‐2 has exploited the mTOR pathway, inhibiting autophagy and so enhancing viral replication.^8^ The anti‐viral activity of SF2523 against the wildtype SARS‐CoV‐2 and multiple VOCs was investigated in a variety of cell lines, including UNCN1T (a bronchial epithelial cell line), Vero STAT1 KO, and Calu‐3 cells, according to the study's authors. Synergistic, anti‐viral effects can be achieved by combining multiple medications with diverse mechanisms of action. Also presented and assessed were a variety of fixed‐dose combinations of SF2523 and either remdesivir (RDV) or MU‐UNMC‐2. The dose‐response per cent inhibition matrix of SF2523/RDV and SF2523/MU‐UNMC‐2 single and combination treatments were also presented and evaluated. SF2523, in combination with other mTOR, PI3K, and BRD4 pathway inhibitors, may hold the key to developing a more effective COVID‐19 treatment.  Given the increasing number of SARS‐CoV‐2 cases and the decreased effectiveness of vaccination‐induced immunity, it is critical to investigate medications that impede the spread of the SARS‐CoV‐2 virus. Rapamycin, for example, is a drug that precisely targets the mTOR signalling system. Rapamycin, along with other autophagy‐inducing treatments such as metformin, statins, and carbamazepine, has been demonstrated to have antiviral properties in laboratory animals.^9^ Molnupiravir and PAXLOVID, which were newly approved, are still VOC sensitive.^10^ A notable feature of this potential SARS‐CoV‐2 therapy is that it targets a variety of host‐virus interactions, notably the mTOR pathway, that are critical to the virus's ability to replicate. Therefore, SF2523 is a promising possible candidate medication for the treatment of COVID‐19 that is currently under investigation. To have a more thorough knowledge of this novel treatment, further research into the mechanisms by which SF2523 inhibits the suppression of autophagy is required.

Overall, drugs that modulate autophagy, such as antioxidants and new compounds, are promising antiviral candidates, and autophagy‐promoting agents such as SF2523 may be regarded as prospective treatments. Further investigation into the exact mechanistic effects of these medications will determine whether they are safe for the general public and successful in inhibiting the replication of SARS‐CoV‐2 in people. Additionally, a thorough understanding of the mechanistic function of autophagy during SARS‐CoV‐2 infection in the elderly remains unknown, and further research is needed.

## CONFLICT OF INTEREST

The authors declare no conflict of interest.
